# The Cytoprotective Effect of C60 Derivatives in the Self-Microemulsifying Drug Delivery System against Triptolide-Induced Cytotoxicity In Vitro

**DOI:** 10.3390/molecules29174073

**Published:** 2024-08-28

**Authors:** Beihua Xu, Zhenyu Wang, Huimin Zhang, Xiao Xu, Mengjie Tang, Gang Wang, Zhongpeng Ding, Ruihao Yu, Meihong Ding, Ting Zhang, Senlin Shi

**Affiliations:** College of Pharmaceutical Sciences, Zhejiang Chinese Medical University, Hangzhou 311400, China; xvbeihua@163.com (B.X.); wzyzcmu@163.com (Z.W.); zhanghuimin202203@163.com (H.Z.); xuxiao0575@163.com (X.X.); tang774124625@163.com (M.T.); 17758035121@163.com (G.W.); 13839710403@163.com (Z.D.); 18058391758@163.com (R.Y.); dingmeihong0125@163.com (M.D.); zhangting55@zcmu.edu.cn (T.Z.)

**Keywords:** triptolide, attenuate, C60-SMEDDS, hepatotoxicity

## Abstract

Objective: The aim of this study was to optimize the formulation of a C60-modified self-microemulsifying drug delivery system loaded with triptolide (C60-SMEDDS/TP) and evaluate the cytoprotective effect of the C60-SMEDDS/TP on normal human cells. Results: The C60-SMEDDS/TP exhibited rapid emulsification, an optimal particle size distribution of 50 ± 0.19 nm (PDI 0.211 ± 0.049), and a near-neutral zeta potential of −1.60 mV. The release kinetics of TP from the C60-SMEDDS/TP exhibited a sustained release profile and followed pseudo-first-order release kinetics. Cellular proliferation and apoptosis analysis indicated that the C60-SMEDDS/TP (with a mass ratio of TP: DSPE-PEG-C60 = 1:10) exhibited lower toxicity towards L02 and GES-1 cells. This was demonstrated by a higher IC50 (40.88 nM on L02 cells and 17.22 nM on GES-1 cells) compared to free TP (21.3 nM and 11.1 nM), and a lower apoptosis rate (20.8% on L02 cells and 26.3% on GES-1 cells, respectively) compared to free TP (50.5% and 47.0%) at a concentration of 50 nM. In comparison to the free TP group, L02 cells and GES-1 cells exposed to the C60-SMEDDS/TP exhibited a significant decrease in intracellular ROS and an increase in mitochondrial membrane potential (ΔψM). On the other hand, the C60-SMEDDS/TP demonstrated a similar inhibitory effect on BEL-7402 cells (IC50 = 28.9 nM) and HepG2 cells (IC50 = 107.6 nM), comparable to that of the free TP (27.2 nM and 90.4 nM). The C60-SMEDDS/TP group also exhibited a similar intracellular level of ROS and mitochondrial membrane potential compared to the SMEDDS/TP and free TP groups. Method: Fullerenol-Grafted Distearoyl Phosphatidylethanolamine-Polyethylene Glycol (DSPE-PEG-C60) was synthesized and applied in the self-microemulsifying drug delivery system. The C60-SMEDDS/TP was formulated using Cremophor EL, medium-chain triglycerides (MCT), PEG-400, and DSPE-PEG-C60, and loaded with triptolide (TP). The toxicity and bioactivity of the C60-SMEDDS/TP were assessed using normal human liver cell lines (L02 cells), normal human gastric mucosal epithelial cell lines (GES-1 cells), and liver cancer cell lines (BEL-7402 cells and HepG2 cells). The production of reactive oxygen species (ROS) after the C60-SMEDDS/TP treatment was assessed using 2′,7′-dichlorofluorescein diacetate (DCFDA) staining. The alterations in mitochondrial membrane potential (ΔψM) were assessed by measuring JC-1 fluorescence. Conclusions: The cytoprotection provided by the C60-SMEDDS/TP favored normal cells (L02 and GES-1) over tumor cells (BEL-7402 and HepG2 cells) in vitro. This suggests a promising approach for the safe and effective treatment of TP.

## 1. Introduction

Tripterygium wilfordii Hook F. is a traditional Chinese herbal medicine. Its crude extracts, known as Tripterygium Glycosides, have been extensively utilized in China for various medical conditions such as rheumatoid arthritis, IgA nephrophy, and lupus nephritis. Triptolide (TP) is the primary active component of Tripterygium Glycosides, which has been extensively studied for its various biological activities, including anticancer [1,2,3], anti-rheumatoid [4], anti-inflammatory [5,6], and immunomodulatory [7,8] effects. In China, Triptolide has been traditionally used for the treatment of psoriasis through transdermal administration, specifically in the form of Triptonide Ointment.

Although triptolide exhibits a variety of pharmacological effects, its clinical application has been limited because of its significant toxicity. It has been reported that the main issue with TP is its toxicity to the digestive system, which leads to liver injury by triggering oxidative stress in hepatocytes [9]. Upstream mechanism studies showed that TP inhibits the mitochondrial respiratory chain, leading to the concentration-dependent accumulation of ROS and oxidative stress. In a study on triptolide-induced liver damage in rats, Fu et al. [10] suggested that TP caused mitochondrial dysfunction, leading to hepatocyte lipid peroxidation by inducing mitochondrial swelling and permeability in the liver mitochondria of female rats. In vitro experiments indicated that triptolide caused an increase in ROS production, a decrease in mitochondrial depolarization, mitochondrial fragmentation, and disturbance in mitochondrial dynamics in L02 cells [11,12].

Fullerenol, a water-soluble derivative of fullerene, has been reported to exhibit strong antioxidant activity, similar to fullerene. It has been reported that fullerenol could act as a free radical scavenger in biological systems and combat oxidative stress from various triggers. Research has shown that fullerenol exhibits protective effects against doxorubicin-induced cytotoxicity in the lungs, kidneys, liver, and testes of rats [13,14,15,16,17]. In previous studies, it was found that C60-modified micelles can protect normal cell lines (L02, H9c2, GES-1) from the cytotoxic effects of anti-tumor drugs, such as doxorubicin and norcantharidin, by counteracting their induced oxidative stress response and reducing intracellular ROS levels in these cells [18,19]. However, the question still remains as to whether fullerenol derivatives could exert protective effects in more complex drug delivery systems such as microemulsions.

Self-microemulsifying drug delivery systems (SMEDDSs), which consist of drugs, oils, surfactants, and cosurfactants, are a crucial strategy for enhancing the bioavailability of poorly water-soluble drugs [20,21]. Oil-in-water (O/W) microemulsions can form with gentle agitation in an aqueous medium, resulting in droplets smaller than 100 nm. The primary advantages of SMEDDSs are their stability and simple formulation processes, which have great potential in pharmaceutical applications and allow for easy industrial scale-up [21,22,23]. Several microemulsions have reached the clinical stage, either in clinical trials or through approval for human use. Examples include Neoral^®^ (cyclosporine capsules), Restasis^®^ (cyclosporine ophthalmic), and Microfol (propofol nanoemulsion for injection, currently in clinical trials).

Since TP induces high levels of ROS in normal tissues, a fullerenol derivative of distearyl phosphatidylethanolamine-polyethylene glycol (DSPE-PEG-C60) was incorporated into the drug delivery system in this study. This derivative was synthesized using our previously established method [18] and was employed as one of the materials for drug carriers. The formulation and technology for the C60-modified TP-loaded SMEDDS (C60-SMEDDS/TP) were optimized. The cytoprotective effects on normal cells (L02 and GES-1) and the cytotoxicity on tumor cells (BEL 7402 and HepG2) of the C60-SMEDDS/TP were further investigated in this study.

## 2. Results

### 2.1. HPLC Analysis of TP

**Figure 1:**
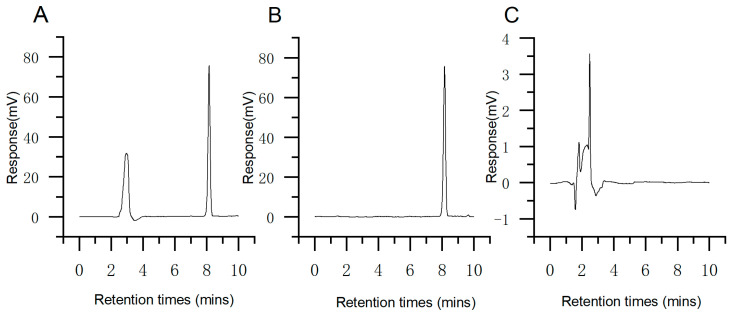
The HPLC spectra of the C60-SMEDDS/TP (**A**); TP (**B**); and C60-SMEDDS (**C**).

The HPLC method was established to determine TP. The C60-SMEDDS/TP solution, the TP solution, and the C60-SMEDDS solution were tested using the chromatographic conditions described in Section 4.2. The results showed that the retention period of TP was approximately 8.11 min, and DSPE-PEG-C60 or excipients did not interfere with the detection of TP (Figure 1A–C). The TP regression curve exhibited a strong linear relationship between 5 μg/mL and 60 μg/mL, with a correlation coefficient (R^2^) exceeding 0.9996.

### 2.2. Preparation and Optimization of the TP-Loaded C60-SMEDDS

#### 2.2.1. GPC Analysis of DSPE-PEG-C60

**Figure 2:**
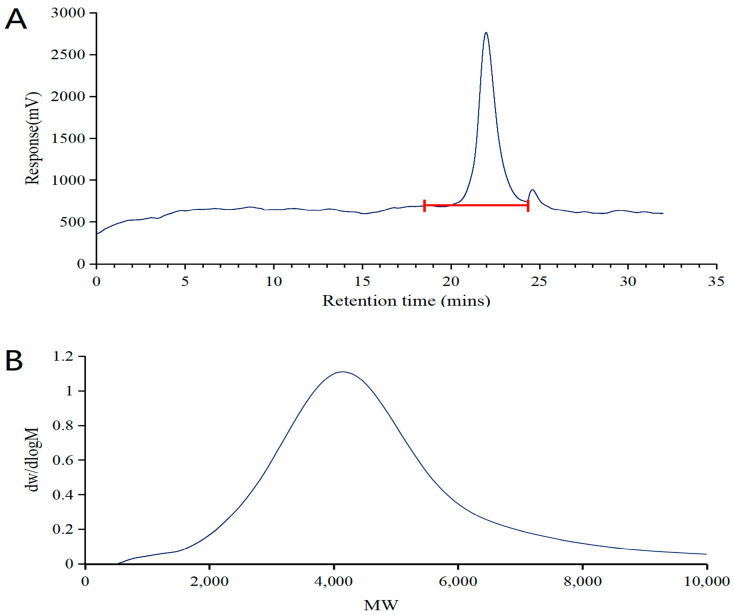
GPC chromatograms of the synthesized copolymer: the retention time of DSPE-PEG-C60 (**A**); the relative molecular weight of DSPE-PEG-C60 (**B**).

The molecular weight of the product was determined by GPC. Figure 2A indicates that the synthesized copolymer was relatively pure, with a larger peak at 22 min, which is the possible product, and a smaller one at 25 min, which is the possible raw material for DSPE-PEG-NH_2_. The elute time had a negative correlation with the size of the polymer. Figure 2B shows the presence of only one peak, indicating the narrow distribution of molecular weight in the synthesized copolymer.

Combined with the information from the previous study, which showed the combination of C60(OH)_22_ and DSPE-PEG-NH2 in the DSPE-PEG-C60 by 1H NMR spectra and the successful coupling of the carbon–carbon double bond of C60(OH)_22_ with the amine group of DSPE-PEG-NH2 by FT-IR spectrum, the synthesized product’s structure can be confirmed as DSPE-PEG-C60.

#### 2.2.2. Pseudo-Ternary Phase Diagram

**Figure 3:**
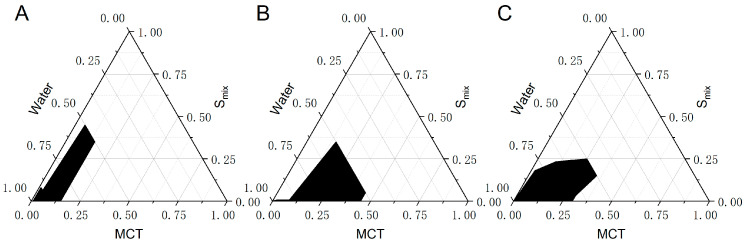
Pseudo-ternary phase diagrams using different ratios of EL/PEG 400 (Smix): Km = 1:1 (**A**); Km = 2:1 (**B**); Km = 3:1 (**C**).

Based on our initial research, we have chosen medium-chain triglycerides (MCT) as the oil, Cremophor EL (EL) as the emulsifier, and PEG 400 as the co-emulsifier. Pseudo-ternary phase diagrams were drawn to determine the optimal conditions for emulsification (Figure 3). The phase diagram was defined by three vertices, namely the oil phase, Smix phase (which consists of a mixture of surfactant and cosurfactant), and water phase. Various ratios of surfactant and cosurfactant mixtures (3:1, 2:1, 1:1) were prepared. Mixtures of oil and Smix in varying ratios, ranging from 1:9 to 9:1, were prepared and subjected to a gradual titration with pure water. The experimental points that can form clear or milky light were designated as prescription points in the pseudo-ternary phase diagram, which can generate self-microemulsions. The enclosed area of the self-emulsifying region was formed by connecting these points with the vertex of the aqueous phase.

According to the pseudo-ternary phase diagram, different proportions of the emulsifier and co-emulsifier corresponded to the formation of a self-emulsifying region of 4.7% (Km = 3:1), 7.2% (Km = 2:1), and 6.4% (Km = 1:1), respectively. These results show that the self-emulsifying region was the largest when the surfactant to co-surfactant ratio was 2:1. Therefore, a Km ratio of 2:1 was selected in the follow-up study for optimization.

#### 2.2.3. Preparation of the TP-Loaded SMEDDS

The experimental design, along with the results for appearance, self-emulsification time, and average particle sizes, is presented in Table 1. The results show that Formulation 2 exhibited the most desirable characteristics, including good transmittance, an ideal droplet size of 50 nm, and rapid self-emulsification when subjected to dilution with mild agitation, out of the SMEDDS formulations prepared. Therefore, the final composition of the C60-SMEDDS/TP consisted of an oil phase (MCT), an emulsifying agent (EL), and a co-emulsifying agent (PEG 400) in a ratio of 1:2:1, loaded with TP (0.2% *W*/*W*) and DSPE-PEG-C60 (2% *W*/*W*).

### 2.3. Characterization of Preparations

#### 2.3.1. Dispersibility Test

**Figure 4:**
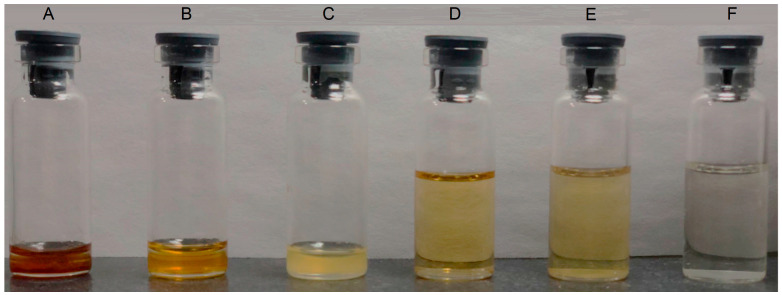
Appearance of the TP-loaded SMEDDS. C60-SMEDDS/TP (DSPE-PEG-C60:TP = 15:1 (*W*/*W*)) (**A**); C60-SMEDDS/TP (DSPE-PEG-C60:TP = 10:1 (*W*/*W*)) (**B**); SMEDDS/TP (DSPE-PEG:TP = 10:1 (*W*/*W*)) (**C**); 100-fold dilution of C60-SMEDDS/TP (DSPE-PEG-C60:TP = 15:1 (*W*/*W*)) (**D**); 100-fold dilution of C60-SMEDDS/TP (DSPE-PEG-C60:TP = 10:1 (*W*/*W*)) (**E**); 100-fold dilution of SMEDDS/TP (DSPE-PEG:TP = 10:1 (*W*/*W*)) (**F**).

The dispersibility test showed that the C60-SMEDDS/TP, when diluted with 0.1 mol·L^−1^ hydrochloric acid at a ratio of 1:100, formed a pale brown microemulsion (Figure 4D,E), while the SMEDDS/TP exhibited a light color (Figure 4F).

#### 2.3.2. Analysis of Droplet Size and Zeta Potential

Droplet size distribution is a critical factor in evaluating a self-microemulsion system. Dynamic light scattering (DLS) is commonly used to measure particle size, zeta potential, and polydispersity index. The particle sizes of the TP-loaded SMEDDS diluted with 0.1 mol·L^−1^ hydrochloric acid were measured to be 50 nm (DSPE-PEG-C60: TP = 10:1 (*W*/*W*)) for the C60-SMEDDS/TP and 69 nm (DSPE-PEG: TP = 10:1 (*W*/*W*)) for the SMEDDS/TP. The particle size was 187 nm for the SMEDDS/TP without the addition of DSPE-PEG-C60 or DSPE-PEG (Table 2). This study showed that the introduction of DSPE-PEG or DSPE-PEG-C60 influenced particle size, resulting in a much smaller microemulsion, while the increase in the amount of DSPE-PEG-C60 or DSPE-PEG within this range had little effect on the particle sizes of the microemulsion.

#### 2.3.3. Morphological Analysis

**Figure 5:**
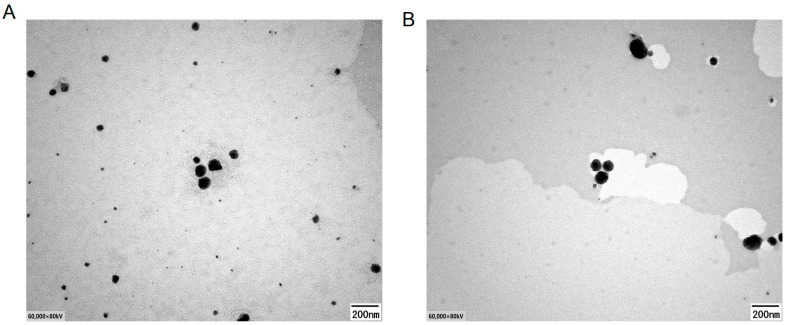
Morphology of the SMEDDS under transmission electron microscope. SMEDDS/TP (DSPE-PEG: TP = 10:1 (*W*/*W*)) (**A**); C60-SMEDDS/TP (DSPE-PEG-C60:TP = 10:1 (*W*/*W*)) (**B**).

The TP-loaded SMEDDS demonstrated microemulsion properties when diluted with 0.1 mol/L hydrochloric acid at a ratio of 1:100 (*W*/*W*). A transmission electron microscopy image is depicted in Figure 5. Through TEM images, it can be observed that most microemulsions are spherical or quasi-spherical. The particle sizes were consistent with the results obtained from DLS.

#### 2.3.4. Drug Release Assay

**Figure 6:**
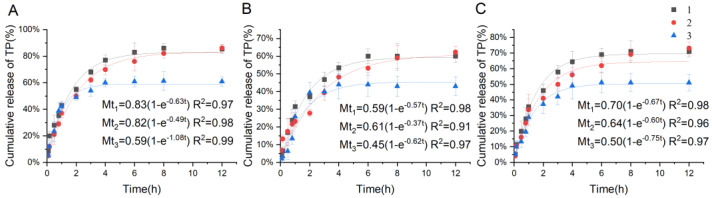
The cumulative release kinetics of TP from the SMEDDS and the pseudo-first-order equation. pH 1.2 artificial gastric juice (**A**); pH 6.8 artificial intestinal fluid (**B**); pH 7.4 phosphate buffer (**C**). (1) SMEDDS/TP; (2) C60-SMEDDS/TP; (3) TP. Data represent the mean ± SD from three independent experiments.

As shown in Figure 6, the release of TP from the SMEDDS formulation followed pseudo-first-order release kinetics. The in vitro release experiments demonstrated that the SMEDDS groups significantly increased the release of TP compared to the raw TP drug. It also indicated that the introduction of C60 had little impact on the release rates of TP from the C60-SMEDDS/TP compared to the SMEDDS/TP. After 12 h, the cumulative release rates of TP from the C60-SMEDDS/TP were observed to be 86% in artificial gastric juice (pH 1.2) and 62% in artificial intestinal fluid (pH 6.8). These rates exceeded those of the raw TP group, which exhibited only 61% release in artificial gastric juice and 43% release in artificial intestinal fluid at the end of 12 h, showing a significant statistical difference (*p* < 0.01). This phenomenon could be explained by the fact that nanoscale oil droplets offer a larger interface surface area for drug release. This leads to the rapid digestion of the microemulsions and the subsequent formation of mixed micelles to solubilize TP released from the drug delivery system. This characteristic will enhance the bioavailability of the C60-SMEDDS/TP, as it will be rapidly and extensively digested within the small intestine.

### 2.4. In Vitro Cytotoxicity Examination

**Figure 7:**
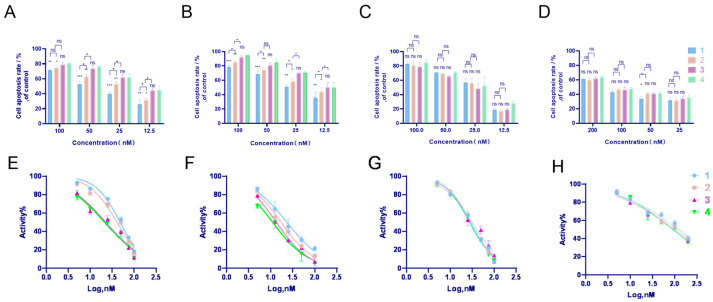
In vitro cytotoxicity of the C60-SMEDDS/TP, the SMEDDS/TP, and free TP was determined after 48 h using CCK-8 assays to measure cell inhibition rates. (**A**,**E**) L02 cell; (**B**,**F**) GES-1 cell; (**C**,**G**) BEL-7402 cell; and (**D**,**H**) HepG2 cell. (1) C60-SMEDDS/TP (DSPE-PEG-C60:TP = 15:1 (*W*/*W*)), (2) C60-SMEDDS/TP (DSPE-PEG-C60:TP = 10:1 (*W*/*W*)), (3) SMEDDS/TP (DSPE-PEG:TP = 10:1 (*W*/*W*)), and (4) free TP. Data represent the mean ± SD from three independent experiments (ns > 0.05, * *p* < 0.05, ** *p* < 0.01, and *** *p* < 0.001).

The cytotoxicity of the C60-SMEDDS/TP was compared to that of free TP and the SMEDDS/TP on L02, GES-1, BEL-7402, and HepG2 cell lines. As shown in Figure 7, there were no significant differences in the cell inhibition rate between the SMEDDS/TP group and the free TP group. However, the C60-SMEDDS/TP exhibited less toxicity with higher IC50 concentrations (40.88 nM on L02 cells and 17.22 nM on GES-1 cells) than the free TP (21.28 nM on L02 cells and 11.09 nM on GES-1 cells) and the SMEDDS/TP (22.69 nM on L02 cells and 13.50 nM on GES-1 cells) when the ratio of DSPE-PEG-C60/DSPE-PEG to TP was 10:1 (*W*/*W*). This study also showed that a higher concentration of C60(OH)_22_ in the delivery system of the C60-SMEDDS/TP (DSPE-PEG-C60:TP = 15:1 (*W*/*W*)) resulted in a slightly more favorable outcome in reducing toxicity (48.09 nM on L02 cells and 23.32 nM on GES-1 cells).

On the other hand, the C60-SMEDDS/TP did not significantly reduce the inhibitory effect on the tumor cells of HepG2 and BEL-7402 compared to the free TP and the SMEDDS/TP.

### 2.5. Detection of Cell Apoptosis

**Figure 8:**
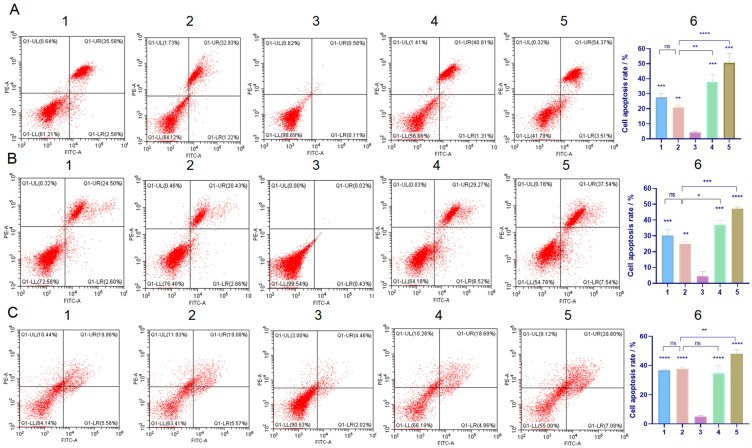
Effects of the TP-loaded SMEDDS and free TP on apoptosis of L02 cells, GES-1 cells, and BEL-7402 cells. Cells were treated with the SMEDDS or free TP at equivalent concentrations of 50 nM TP (L02 cells and GES-1 cells) or 75 nM TP (BEL-7402 cells) for 24 h, followed by Annexin V/PI staining and flow cytometry detection. (**A**) Representative pseudo-color plots of Annexin V/PI staining of L02 cells and the apoptosis ratio of the calculated L02 cells. (**B**) Representative pseudo-color plots of GES-1 cells and the apoptosis ratio of the calculated GES-1 cells. (**C**) Representative pseudo-color plots of BEL-7402 cells and the apoptosis ratio of the calculated BEL-7402 cells. (1) C60-SMEDDS/TP (DSPE-PEG-C60:TP = 15:1 (*W*/*W*)), (2) C60-SMEDDS/TP (DSPE-PEG-C60:TP = 10:1 (*W*/*W*)), (3) Control, (4) SMEDDS/TP (DSPE-PEG:TP = 10:1 (*W*/*W*)), (5) free TP, and (6) the apoptosis ratio of the calculated cells. The values presented are the means ± standard deviations of three independent experiments (ns > 0.05, * *p* < 0.03, ** *p* < 0.01, *** *p* < 0.001, **** *p* < 0.0001).

An apoptosis assay was conducted to confirm the protective effect of C60 derivatives on normal cell lines. As depicted in the representative pseudo-color plots and the apoptosis ratios of the cells (Figure 8), the SMEDDS/TP group exhibited decreased apoptosis rates compared to the free TP group. This may be due to the incomplete release of TP from microemulsion during testing [24]. The C60-SMEDDS/TP group showed a more significant decrease in apoptosis rate in L02 cells and GES-1 cells compared to the SMEDDS/TP group. This was demonstrated by a decreased apoptosis ratio of 20.87% (L02) and 26.3% (GES-1) for the C60-SMEDDS/TP (DSPE-PEG-C60:TP = 10:1 (*W*/*W*)), compared to the apoptosis rate observed in the free TP group (50.6% and 47.2%) and the SMEDDS/TP group (34.8% and 33.9%). However, the increased concentration of C60(OH)_22_ in the delivery system within this range did not result in a further decrease in the apoptosis rate.

On the other hand, the C60-SMEDDS/TP exhibited similar cytotoxicity (37.8%) against BEL-7402 cells compared to the SMEDDS/TP (36.0%). This finding was consistent with the results of the CCK-8 assay.

### 2.6. Evaluation of Intracellular Levels of ROS

**Figure 9:**
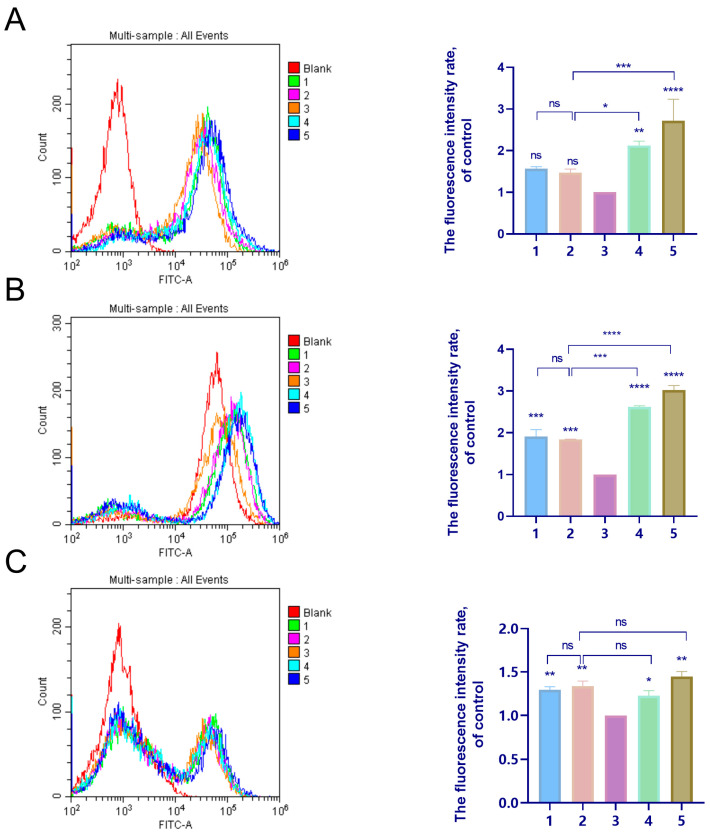
Effects of the TP-loaded SMEDDS and free TP on ROS levels in L02 cells, GES-1 cells, and BEL-7402 cells. Cells were treated with the TP-loaded SMEDDS or free TP at equivalent concentrations of 50 nM TP (L02 cells and GES-1 cells) or 75 nM TP (BEL-7402 cells) for 24 h, followed by DCFDA staining and flow cytometry detection. (**A**) Representative fluorescence intensity plots of DCF in L02 cells, and the fluorescence intensity rate of the calculated L02 cells. (**B**) Representative fluorescence intensity plots of DCF in GES-1 cells, and the fluorescence intensity rate of the calculated GES-1 cells. (**C**) Representative fluorescence intensity plots of DCF in BEL-7402 cells, and the fluorescence intensity rate of the calculated BEL-7402 cells. (1) C60-SMEDDS/TP (DSPE-PEG-C60:TP = 15:1 (*W*/*W*)), (2) C60-SMEDDS/TP (DSPE-PEG-C60:TP = 10:1 (*W*/*W*)), (3) Control, (4) SMEDDS/TP (DSPE-PEG:TP = 10:1 (*W*/*W*)), and (5) free TP. The values presented are the means ± standard deviations of three independent experiments (ns > 0.05, * *p* < 0.03, ** *p* < 0.01, *** *p* < 0.001, **** *p* < 0.0001).

ROS levels in cell lines treated with the TP-loaded SMEDDS or the free TP were measured using flow cytometry with DCFDA staining. Compared to the control group, the group treated with free TP showed a significant increase in the level of ROS in normal cells, with a fold change of 2.72 in L02 cells and 2.95 in GES-1 cells (Figure 9A,B). The groups treated with the C60-SMEDDS/TP showed a decrease in intracellular ROS generation. The DCF level of the C60-SMEDDS/TP (DSPE-PEG-C60:TP = 10:1 (*W*/*W*)) was approximately 1.47-fold in L02 cells and 1.82-fold in GES-1 cells. These levels were lower than those of the free TP group and the SMEDDS/TP group (2.12 in L02 cells and 2.55-fold in GES-1 cells). There was no significant difference between the C60-SMEDDS/TP groups with different amounts of C60(OH)_22_ in the delivery system.

ROS levels were also assessed in BEL-7402 cells following treatment with the TP-loaded SMEDDS or free TP (Figure 9C). The fluorescence levels observed in the C60-SMEDDS/TP groups did not show a significant difference when compared to the SMEDDS/TP group.

### 2.7. Cell Mitochondrial Function Evaluation

**Figure 10:**
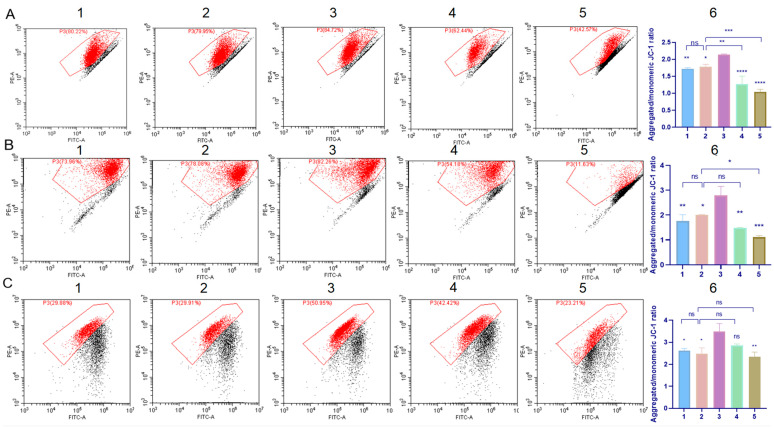
Effects of the TP-loaded SMEDDS and free TP on the mitochondrial membrane potential of L02 cells, GES-1 cells, and BEL-7402 cells. Cells were treated with the TP-loaded SMEDDS or free TP at equivalent concentrations of 50 nM (L02 cells, GES-1 cells) or 75 nM TP (BEL-7402 cells) for 24 h. Mitochondrial membrane potential (ΔψM) was measured by JC-1 staining, followed by flow cytometry. (**A**) Representative pseudo-color plots of JC-1 staining of L02 cells and graphs represent the ratio of red fluorescence (JC-1 aggregates) to green fluorescence (JC-1 monomers) compared to the control. (**B**) Representative pseudo-color plots of JC-1 staining of GES-1 cells and graphs represent the ratio of red fluorescence (JC-1 aggregates) to green fluorescence (JC-1 monomers) compared to the control. (**C**) Representative pseudo-color plots of JC-1 staining of BEL-7402 cells and graphs represent the ratio of red fluorescence (JC-1 aggregates) to green fluorescence (JC-1 monomers) compared to the control. (1) C60-SMEDDS/TP (DSPE-PEG-C60:TP = 15:1 (*W*/*W*)), (2) C60-SMEDDS/TP (DSPE-PEG-C60:TP = 10:1 (*W*/*W*)), (3) Control, (4) SMEDDS/TP (DSPE-PEG:TP = 10:1 (*W*/*W*)), (5) free TP, and (6) the ratio of red fluorescence (JC-1 aggregates) to green fluorescence (JC-1 monomers) compared to the control cells. The values presented are the means ± standard deviations of three independent experiments (ns > 0.05, * *p* < 0.03, ** *p* < 0.01, *** *p* < 0.001, **** *p* < 0.0001).

The changes in mitochondrial membrane potential were assessed using JC-1 staining. As shown in Figure 10, the TP treatment led to a reduction in JC-1 aggregates in cells. This decrease was accompanied by a reduced red-to-green fluorescence ratio, which was approximately 1.04-fold in L02 cells and 1.12-fold in GES-1 cells compared to the control group (2.13-fold and 2.79-fold). This indicates a significant impairment of mitochondrial function induced by TP. At the same time, the C60-SMEDDS/TP (DSPE-PEG-C60:TP = 10:1 (*W*/*W*)) group exhibited higher ratios of about 1.78-fold in L02 cells and 1.97-fold in GES-1 cells compared to the SMEDDS/TP group (1.28-fold and 1.52-fold). These results confirm that the C60-modified SMEDDS had a protective effect against TP-induced damage.

On the other hand, the presence of C60 derivative in the SMEDDS did not change the effect of TP on BEL-7402 cells compared to the free TP group and the SMEDDS/TP group.

## 3. Discussion

Triptolide (TP) demonstrates a variety of biological activities and pharmaceutical effects. However, the toxicity of TP to the alimentary system, as well as its poor solubility and low bioavailability, limits its wide clinical application. Numerous studies have reported different delivery systems for the targeted administration of TP to tumors or inflamed tissue. The enhanced permeability and retention (EPR) effect of solid tumors and inflammatory tissue has been extensively utilized in the field of nanomedicine. However, achieving full drug enrichment in tumors or other targeted locations is a challenging task. Furthermore, TP aims to produce systemic effects in order to achieve therapeutic outcomes. Reducing cytotoxicity to normal tissues is an important strategy for minimizing the side effects of drugs. C60(OH)n has been reported to possess antioxidant properties, offering protection against cytotoxicity induced by DOX. A derivative of C60(OH)n (DSPE-PEG-C60) developed by our team was found to exhibit cytoprotective effects against DOX-induced damage in normal cells in vitro, as demonstrated in our previous study [18]. DSPE-PEG-C60 was also found to be compatible with the oil phase in this study. Then, the amphiphilic DSPE-PEG-C60 was used as one of the components in formulating drug carriers for the development of the C60-SMEDDS/TP. The inclusion of DSPE-PEG-C60 in the SMEDDS led to the production of nanoparticles measuring 50 nm in size with a near-neutral zeta potential of −1.60 mV (Table 2).

Oral absorption of microemulsions is important for the in vivo function of C60-modified microemulsions. Researchers investigated the in vivo absorption of microemulsions following oral administration through a visual study. According to their imaging data, smaller particles (50–100 nm) were taken up by enteric epithelial cells more readily than larger particles (500–1000 nm), which were rapidly digested and formatted into mixed micelles. Some microemulsions are carried by the lymphatics after being absorbed by the epithelium, which greatly enhances their bioavailability and overall absorption. This transport mechanism helps avoid first-pass hepatic metabolism [25,26]. According to the above results, our specially designed C60-SMEDDS/TP with their appropriate particle sizes would be expected to enhance intestinal absorption in vivo.

Reactive oxygen species (ROS), which cause DNA breakage and lipid peroxidation of the cell membrane, are believed to be involved in the mechanism of TP-induced cell damage [10,11,12]. Considering the critical role that ROS-mediated inflammation plays in the pathogenesis of TP, we investigated the potential protective effects of the C60-modified SMEDDS against TP-induced cellular ROS. Compared to free TP and the SMEDDS/TP, the C60-SMEDDS/TP can more effectively scavenge free radicals, reduce intracellular ROS, and decrease the apoptosis of L02 and GES-1 cells. This study also investigated the protective effects of the C60-SMEDDS/TP on mitochondrial membrane potential during TP-induced injury. The results showed that the DSPE-PEG-C60/TP group had a greater mitochondrial membrane potential than the free TP group and the SMEDDS/TP group on L02 and GES-1 cells. The cytoprotective effects of the C60-SMEDDS/TP on normal cell lines are consistent with our earlier findings [18,19].

Would the therapeutic efficacy of TP in combating cancer be compromised when the C60-SMEDDS/TP is protecting the viability of healthy cells? Our study found that, unlike previous studies, DSPE-PEG-C60 showed a slight compromise in antitumor ability compared to the TP group on BEL-7402 and HepG2 cells, as demonstrated in the apoptosis tests. However, this compromise would be weakened when drug exposure time was prolonged, as demonstrated in the CCK-8 tests. Given that liver damage is the primary adverse reaction of TP, the use of the C60-SMEDDS/TP could potentially offer a more favorable safety profile.

## 4. Materials and Methods

### 4.1. Materials

TP (Triptolide, CAS: 38748-32-2, Shanghai YiEn Chemical Technology Co., Ltd., Shanghai, China), fullerenol (C60(OH)_22_, Hengqiu Technology Co., Ltd., Suzhou, China), DSPE-PEG(2000)-NH_2_ (CAS: 474922-26-4, Yuxi Pharmaceutical Technology Co., Ltd., Yuxi, China), medium-chain triglyceride (MCT, CAS: 538-24-9, Shanghai Aladdin Biochemical Technology Co., Ltd., Shanghai, China), Cremophor EL (EL, CAS: 61791-16-2, Shanghai Aladdin Biochemical Technology Co., Ltd., Shanghai, China), and PEG 400 (CAS: 25322-68-3, Shanghai Bailingwei Chemical Technology Co., Ltd., Shanghai, China). Cell Counting Kit-8 (CCK-8, Abbkine Scientific Co., Ltd., Wuhan, China), Annexin V-FITC/PI Apoptosis Assay Kit (Biosharp Biology Co., Ltd., Hangzhou, China), JC-1 (Biosharp Biology Co., Ltd., Hangzhou, China), Reactive Oxygen Species Assay Kit (Biosharp Biology Co., Ltd., Hangzhou, China), Fetal Bovine Serum (FBS) (Thermo Fisher Scientific, Shanghai, China), DMEM/RPMI 1640 (Thermo Fisher Scientific, Shanghai, China), and Penicillin-streptomycin Double Resistance (Biosharp Biology Co., Ltd., Hangzhou, China) were used in this study.

### 4.2. Determination of TP Content

Chromatographic conditions: Diamonsil C18 (4.6 mm × 150 mm, 5 μm); mobile phase: methanol–water (V:V = 40:60); flow rate: 1 mL·min^−1^; detection wavelength: 218 nm; injection volume: 10 μL; column temperature: 30 °C. Preparation of TP standard solution: 10 mg of TP reference was accurately weighed in a 10 mL volumetric flask, dissolved in methanol, and diluted to create a stock solution with a concentration of 1 g·L^−1^. The solution was then stored at 4 °C. A proper amount of the stock solution was taken and diluted into a series of standard solutions using methanol. The standard curve equation was derived through a linear regression analysis, with the peak area (A) as the dependent variable and the mass concentration of TP (ρ, mg·L^−1^) as the independent variable.

### 4.3. Preparation and Optimization of the TP-Loaded C60-SMEDDS

#### 4.3.1. Synthesis of DSPE-PEG-C60

DSPE-PEG-C60 was prepared based on our previous laboratory research [18]. In short, fullerenol (C60(OH)_22_) and equimolar DSPE-PEG-NH_2_ were put in a round-bottomed flask and dissolved with purified water. The solution was stirred at 50–60 °C for 8–12 h. The simple detection method for determining the reaction endpoint is to add a drop of the reaction solution to ethanol. Complete dissolution indicates that C60(OH)_22_ has been successfully grafted onto the polymer material. If precipitation occurs, it indicates that the reaction endpoint has not been reached, and the ungrafted C60(OH)_22_ precipitates in ethanol. Then, the reaction solution was dialyzed with water (MW = 2000) three times. After freeze-drying, a brown product was obtained with a yield of approximately 90%.

#### 4.3.2. Characterization of DSPE-PEG-C60

GPC measurements were conducted on a GPC system (Waters Ultrahydrogel 300 × 7.8 mm 500-250-120A column, Agilent RID G1362A Detector, Agilent 1260, Santa Clara, CA, USA) using water as the eluent (flow rate: 1 mL/min) and polyethylene glycol was used as standards.

#### 4.3.3. Construction of Pseudo-Ternary Phase Diagram

Pseudoternary phase diagrams were constructed to determine the region of self-emulsification. In this process, visual observation was the primary method of judgment. Emulsifier EL and co-emulsifier PEG400 were combined in mass ratios of 3:1, 2:1, and 1:1. The mixture was then vortexed and mixed for a duration of 3 min, resulting in the formation of a transparent mixture (Smix).

According to the ratios of 9:1, 8:2, 7:3, 6:4, 5:5, 4:6, 3:7, 2:8, and 1:9, the oil phase of MCT (O) and the mixture phase (Smix) were mixed and stirred with a magnetic stirrer for 5 min to form a transparent oil. The oil phase (O) or mixture phase (Smix) was transferred to a beaker, and purified water was added while stirring with a magnetic stirrer at 200 r·min^−1^. The amount of water added was recorded when the solution suddenly changed from clear to turbid liquid, and a pseudo-ternary phase diagram was drawn.

#### 4.3.4. Preparation of the TP-Loaded SMEDDS

After mixing the medium-chain triglyceride (MCT), EL, and PEG 400 in the proportions shown in Table 1, specific amounts of TP (approximately 2 mg·g^−1^ SMEDDS) and suitable DSPE-PEG-C60 (or DSPE-PEG) were added. The weight ratio of DSPE-PEG-C60 (or DSPE-PEG) to TP was fixed at 10:1 or 15:1. The mixture was then agitated in a constant-temperature bath at 37 °C for 24 h.

### 4.4. Characterization of Preparations

#### 4.4.1. Dispersibility Test

The C60-SMEDDS/TP or the SMEDDS/TP concentrate was added to a 0.1 mol/L hydrochloric acid solution at 37 °C in a ratio of 50:1. The mixture was then stirred at a speed of 100 rpm and the emulsion was observed for clarification.

#### 4.4.2. Droplet Size and Zeta Potential

The C60-SMEDDS/TP or the SMEDDS/TP concentrate (1 g) was taken, 0.1 mol/L hydrochloric acid was added to 50 mL at 37 °C, and was stirred evenly at 100 r/min. The Zetasizer Nano ZS-90 (Malvern, UK) was utilized to measure the particle size and zeta potential.

#### 4.4.3. Morphology

The morphology of the C60-SMEDDS/TP or the SMEDDS/TP was examined using an S-4800 scanning electron microscope (Hitachi, Tokyo, Japan). Samples were prepared as previously described (Section 4.3.2). An appropriate amount of microemulsion was applied to the copper mesh, ensuring uniform distribution. Then, a 2.0% solution of phosphotungstic acid was added and the sample was subjected to negative staining for 10 min. Following this, the sample was air-dried at room temperature and subsequently examined using TEM.

#### 4.4.4. Drug Release Study

The dilution ratio was determined within a specific range, with the volume of adult gastric juice being approximately 100 mL and the emulsification time kept below 2 min. Precisely 2 mL of the C60-SMEDDS/TP, the SMEDDS/TP, or an equivalent amount of TP was placed into RC dialysis bags (MW 8000 to 14,000). These bags were then immersed in three different release media: phosphate buffer (pH 7.4, 50 mL), artificial gastric juice (pH 1.2, 50 mL), and artificial intestinal fluid (pH 6.8, 50 mL). The mixture was agitated at 100 rpm/min at 37 °C. At 0.083, 0.16, 0.5, 0.8, 1, 2, 3, 4, 6, 8, and 12 h, dialysates (2 mL each) were taken and the concentration of TP released was measured using HPLC. The rate of TP release from the SMEDDS was calculated by dividing the amount of TP released at a specific time by the initial TP content in the SMEDDS.

### 4.5. Cell Culture

Human hepatocellular carcinoma (HCC) cell lines (BEL-7402, HepG2) and immortalized normal human hepatocytes (L02), immortalized normal human gastric mucosal epithelial cell line (GES-1) were obtained from the Cell Bank of the Chinese Academy of Sciences in Shanghai, China. HepG2 cells, L02 cells and GES-1 were cultured in DMEM medium supplemented with 10% fetal bovine serum (FBS) and 1% antibiotics (100 U/mL penicillin G and 0.1 mg/mL streptomycin). BEL-7402 cells were maintained in RPMI 1640 medium supplemented with 10% FBS and 1% antibiotics (100 U/mL penicillin G and 0.1 mg/mL streptomycin). Cells were cultured at 37 °C in a humidified environment with 5% CO_2_.

### 4.6. CCK-8 Assay

The CCK-8 method was used to detect cell activity. Cells were seeded in a 96-well plate at an initial density of 3 × 10^3^ cells/well. After a 24 h incubation period, the cells were subjected to treatment with the free TP or the TP-loaded SMEDDS for 48 h. Subsequently, 10 μL of CCK-8 reagent was added to each well. The cells were incubated for an additional 30 to 60 min. Every hole’s absorbance value (OD value) was determined at 450 nm using an MK-3 microplate reader (Thermo, Waltham, MA, USA). The default cell activity value for the untreated control samples was 100%. The formula used to calculate the cell inhibition rate was [(OD_450_ control well − OD_450_ administration well)/OD_450_ control well] × 100%.

### 4.7. Apoptosis Detection

Flow cytometry was utilized to evaluate the effect of the C60-SMEDDS/TP on cell apoptosis. Cell cultures were prepared by seeding cells in 6-well plates at a density of 3 × 10^5^ cells per well. The cells were subsequently treated with free TP, the SMEDDS/TP, or the C60-SMEDDS/TP at the same concentration of TP for a duration of 24 h. The concentrations used were 75 nM for GES-1 cells, 50 nM for L02 cells, and 200 nM for HepG2 cells. An Annexin V-FITC Apoptosis Detection kit was utilized in accordance with the manufacturer’s instructions. After completing the staining process, the cell suspension was filtered and subsequently tested. The assessment of cell apoptosis was conducted using flow cytometry (CytoFlEX Beckman, Brea, CA, USA). The FL1 channel was utilized to detect the green fluorescence emitted by Annexin V-FITC, while the FL3 channel was used to detect the red fluorescence emitted by Propidium Iodide (PI). Data were calculated using the CytExpert 2.4 software.

### 4.8. ROS Production Detection

The intracellular accumulation of ROS was assessed using the fluorescent probe DCFDA. Cells were seeded in 6-well plates at a density of 3 × 10^5^ cells per well. Following a 24 h exposure to either free TP or the TP-loaded SMEDDS at an equivalent concentration of TP, the cells were subsequently incubated with the fluorescent probe DCFDA in the dark at 37 °C for 30 min. The cells were collected and the fluorescence intensity was promptly assessed using flow cytometry.

### 4.9. Mitochondria Membrane Potential Determination

The mitochondrial membrane potential was measured using JC-1 fluorescence. Cells were seeded in 6-well plates at a density of 3 × 10^5^ cells/well. After being exposed to either free TP or the TP-loaded SMEDDS at an equivalent concentration for 24 h, the cells were stained with a JC-1 probe at a concentration of 5 μg/mL. The cells were then incubated in a dark environment at 37 °C for 30 min and subjected to three washes using JC-1 buffer. Subsequently, the stained cells were analyzed using flow cytometry to detect fluorescence in the emission ranges of fluorescein isothiocyanate (FITC) for JC-1 monomers and phycoerythrin (PE) for JC-1 aggregates.

### 4.10. Statistical Analysis

All data were generated from three independent experiments and presented as the means ± SD. The data were analyzed using Student’s *t*-test by Prism software version 8.0 (Graph Pad Software Inc., San Diego, CA, USA) and the critical level of significance was set at *p* < 0.05.

## 5. Conclusions

In this study, a prepared C60-SMEDDS/TP exhibited rapid emulsification, had an optimal particle size of 50 nm (PDI 0.211), and a near-neutral zeta potential of −1.60 mV. This study demonstrated that the C60-SMEDDS/TP significantly reduced TP-induced cytotoxicity in normal cells (L02, GES-1). The potential mechanism underlying this phenomenon could involve the scavenging of free radicals, the inhibition of oxidative stress, the restoration of mitochondrial membrane potential, and the inhibition of cell apoptosis in normal cells. The results also showed that the C60-SMEDDS/TP exhibited similar antineoplastic activity to the free TP group in human hepatocellular carcinoma cells (HepG2, BEL-7402) in vitro.

The decrease in cytotoxicity after the formation of the C60-SMEDDS/TP was only observed at the cellular level. The impact of carriers on reducing the toxicity of loaded drugs is typically associated with alterations in circulation and pharmacokinetics. Hence, additional in vivo experiments are necessary to validate the safety of the C60-SMEDDS/TP and elucidate the role and mechanism of toxicity reduction in vital organs. Further investigations are in progress to examine their safety in clinically relevant animal models.

## Figures and Tables

**Table 1:** Formulation optimization results of the C60-SMEDDS/TP (DSPE-PEG-C60:TP = 10:1 (*W*/*W*)).

	MCT (%)	EL (%)	PEG-400 (%)	Self-Emulsifying Time	Appearance	Size (nm)
1	10	60	30	<30 s	Clear	19.14 ± 0.07
2	25	50	25	<30 s	Milky light	50.06 ± 0.19
3	40	40	20	<30 s	Milky light	91.22 ± 1.59
4	55	30	15	<30 s	Milky light	29.85 ± 0.33
5	70	20	10	>30 s	Clear	176 ± 1.25
6	85	10	5	>30 s	Cloudy	441.8 ± 7.0

**Table 2:** Particle size (PS), PDI, and zeta potential (ZP) of the TP-loaded SMEDDS.

	DSPE-PEG-C60:TP = 10:1 (*W*/*W*)	DSPE-PEG-C60:TP = 15:1 (*W*/*W*)	DSPE-PEG:TP = 10:1 (*W*/*W*)	DSPE-PEG:TP = 15:1 (*W*/*W*)	Regular SMEDDS/TP
PS (nm)	50 ± 0.19	50 ± 0.28	69 ± 0.59	68 ± 0.12	187 ± 0.18
PDI	0.21 ± 0.049	0.20 ± 0.008	0.29 ± 0.010	0.29 ± 0.015	0.19 ± 0.015
ZP (mv)	−1.60	−2.81	+1.52	−0.16	+1.24

## Data Availability

The original contributions presented in the study are included in the article, further inquiries can be directed to the corresponding authors.

## Abbreviations

TP	Triptolide
SMEDDS	Self-microemulsifying drug delivery system
O/W	Oil-in-water
MCT	Medium-chain triglycerides
EL	Cremophor EL
ROS	Reactive Oxygen Species
LD_50_	Median lethal dose
EPR	Effect enhanced permeability and retention effect
DSPE-PEG	1,2-Distearoyl-sn-glycero-3-phosphoethanolamine-N-methoxy-[poly(ethylene glycol)]; PEG MW = 2000
C60(OH)_22_	Fullerenol
DSPE-PEG-C60	Fullerenol-Grafted 1,2-Distearoyl-sn-glycero-3-phosphoethanolamine-N-methoxy-[poly(ethylene glycol)]
CCK-8	Cell Counting Kit-8
FITC	3′,6′-dihydroxy-5-isothiocyanato-3H-spiro[isobenzofuran-1,9′-xanthen]-3-one
PI	Propidium iodide
PE	Phycoerythrin
HPLC	High-Performance Liquid Chromatography
TEM	Transmission Electron Microscopy
DLS	Dynamic light scattering
DCFDA	2′,7′-Dichlorodihydrofluorescein diacetate
IC_50_	Half-maximal inhibitory concentration
HCC	Human hepatocellular carcinoma
